# Proteomic analysis of glomeruli, tubules and renal interstitium in idiopathic membranous nephropathy (IMN): A statistically observational study

**DOI:** 10.1097/MD.0000000000036476

**Published:** 2023-12-15

**Authors:** Chang Lu, Zhi-Feng Luo, Donge Tang, Fengping Zheng, Shanshan Li, Shizhen Liu, Jing Qiu, Fanna Liu, Yong Dai, Wei-Guo Sui, Qiang Yan

**Affiliations:** a The Organ Transplantation Department of No.924 Hospital of PLA Joint Logistic Support Force, Medical quality specialty of the Joint Logistic Support Force, Guangxi Key Laboratory of Metabolic Diseases Research, Guilin, Guangxi, P.R. China; b Clinical Medical Research Center, Guangdong Provincial Engineering Research Center of Autoimmune Disease Precision Medicine, Shenzhen Engineering Research Center of Autoimmune Disease, The Second Clinical Medical College of Jinan University, Shenzhen People’s Hospital, Shenzhen, Guangdong, P.R. China; c The Second Department of Urology, Affiliated Hospital of Guilin Medical University, Guilin, Guangxi, P.R. China; d Institute of Nephrology and Blood Purification, the First Affiliated Hospital of Jinan University, Jinan University, Guangzhou, P.R. China.

**Keywords:** idiopathic membranous nephropathy, laser capture microdissection, mass spectrometry, proteomic analysis

## Abstract

Idiopathic membranous nephropathy (IMN) is a common type of primary glomerulonephritis, which pathogenesis are highly involved protein and immune regulation. Therefore, we investigated protein expression in different microregions of the IMN kidney tissue. We used laser capture microdissection and mass spectrometry to identify the proteins in the kidney tissue. Using MSstats software to identify the differently expressed protein (DEP). Gene ontology analysis and Kyoto Encyclopedia of Genes and Genomes pathway analysis were used to predict and enrich the potential functions of the DEPs, and DEPs were compared to the Public data in the gene expression omnibus (GEO) database for screening biomarkers of IMN. Immune infiltration analysis was used to analyze the immune proportion in IMN. Three significantly up-regulated proteins were identified in the glomeruli of patients with IMN; 9 significantly up-regulated and 6 significantly down-regulated proteins were identified in the interstitium of patients with IMN. Gene ontology analysis showed that the DEPs in the glomerulus and interstitium were mostly enriched in “biological regulation, the immune system, and metabolic processes.” Kyoto Encyclopedia of Genes and Genomes analysis showed that the DEPs in the glomerulus and interstitium were mostly enriched in the “immune system” and the “complement and coagulation cascades. ” According to the public information of the GEO database, DEPs in our study, Coatomer subunit delta Archain 1, Laminin subunit alpha-5, and Galectin-1 were highly expressed in the IMN samples from the GEO database; in the immune infiltration analysis, the proportion of resting memory CD4 T cells and activated NK cells in IMN were significantly higher than in the normal group. This study confirmed that there were significant differences in protein expression in different micro-regions of patients with IMN, The protein Coatomer subunit delta Archain 1, Laminin subunit alpha 5, Galectin-1 are potential biomarkers of IMN, the memory T cells CD4 and NK cells, maybe involved in the immunologic mechanism in the development of IMN.

## 1. Introduction

Idiopathic membranous nephropathy (IMN) is one of the most common primary glomerulonephritis causes primary nephrotic syndrome in adults. The main pathological features of IMN include diffuse thickening of the glomerular basement membrane and immune complex deposition in visceral epithelial cells. There is still no effective treatment for IMN because its pathogenesis remains unclear.^[1]^ Several previous studies have shown that podocyte antigens, circulating antibodies, complement system activation, and genetic factors can cause glomerular podocyte dysfunction and proteinuria. These may be considered the most important mechanisms in current research on IMN.^[2–5]^ However, these results still lack sufficient clear evidence for the formation process of IMN These results still lack sufficient clear evidence for the formation of IMN, and could not fully explain the complete pathophysiological process of IMN.

Previous studies have found that microenvironmental regulation of immune compartmentalization might be an important mechanism for the local defense function of the kidney.^[6]^ It is well known that glomerular diseases caused by various reasons are often accompanied by damage to the renal tubules and interstitium, and subsequent renal fibrosis, which is a common pathway from chronic kidney disease to end-stage renal failure. Recent studies have identified 2 novel proteins, exostosin 1 and exostosin 2, in glomeruli separated by laser capture microdissection (LCM) and suggested that they are related to membranous nephropathy.^[7]^ This indicates that immune compartmentalization may play an important role in kidney diseases. However, complete immune compartmentalization, including glomeruli, renal tubules, and renal interstitials, in IMN has not been reported to date.

In this study, we identified several other proteins in the glomeruli, tubules, and interstitium of patients with IMN by using LCM, liquid chromatography (LC), and mass spectrometry (MS).

## 2. Materials and methods

### 2.1. Subjects

Five patients diagnosed with IMN by renal biopsy at the First Affiliated Hospital of Jinan University (Guangzhou, China) and Shenzhen People Hospital (Shenzhen, China) were included in this study. These patients did not undergo any systematic treatment prior to blood, urine and kidney tissues sampling. The kidney tissues are collected by renal puncture. Secondary IMN caused by autoimmune diseases, infectious diseases, and cancer was excluded based on renal biopsy and serological evidence. Patients with other combined kidney diseases, such as diabetic nephropathy, obesity-related nephropathy, and qualitative nephritis, were also excluded. Histological lesions were classified in accordance with the Ehrenreich classification system. In addition, we collected 5 kidney tissues from renal transplantation donors whose diagnosed brain death, with age and sex matched the patients were recruited as the control group. Blood samples, urine samples, and clinical information of all subjects were collected before renal biopsy and stored at −80ºC for later use.

**Figure 1.**
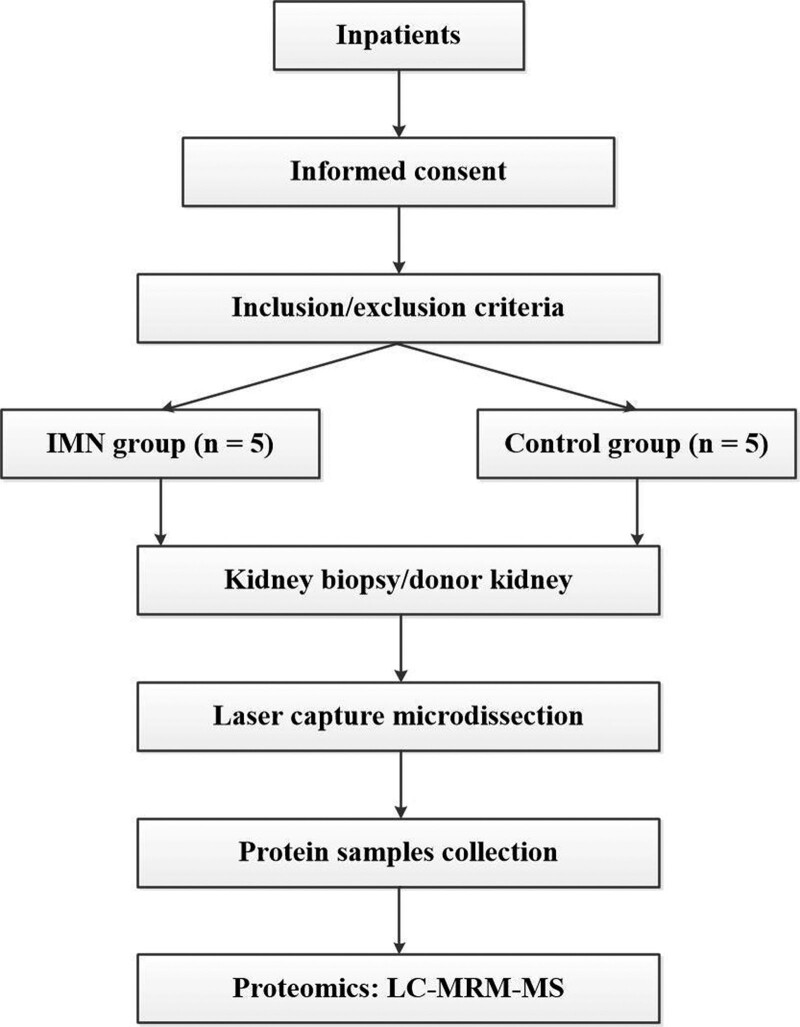
Flowchart of the study.

This study is in line with the purpose of the Declaration of Helsinki and was approved by Guilin No. 924 Hospital. All the study participants and the legal guardian of donors are provided written informed consent. The study protocol was reviewed and approved by Institutional Review Board of the First Affiliated Hospital of Jinan University (approval number [KY-2020-034]). A flowchart of the study is shown in Figure 1.

### 2.2. Laser capture microdissection

The remaining kidney biopsies that were not used for diagnostic tests were fixed with formalin and embedded in paraffin. Ten-micrometer-thick sections were deparaffinized, rehydrated, and stained with hematoxylin and eosin. Glomeruli with global sclerosis, more than minimal segmental sclerosis, crescent formation, fibrinoid necrosis, renal tubules with atrophy and dissolution, and renal interstitium with fibrosis were excluded. According to these standards, laser microdissection (Leica 7000, Germany) of the qualified glomeruli, renal tubules, and renal interstitium was performed and dropped into the test tube cap by gravity. For each patient, we aimed to microdissection approximately 100 glomerular, 200 renal tubule, and renal interstitial cross-sections equivalent to 100 glomeruli. The microdissected glomeruli, renal tubules, and renal interstitium were suspended in 10 μL of lysate and stored at −80ºC until peptide extraction.

### 2.3. Protein extraction

Protein extraction of the glomeruli was performed microscopically, and the renal tubules and interstitium were treated using the same method. Samples were ground into a powder in liquid nitrogen and extracted with lysis buffer containing 1 mM phenylmethylsulfonyl fluoride (PMSF) and 2 mM ethylene diamine tetraacetic acid. After 5 minutes, 10 mM dithiothreitol was added to the samples. The suspension was sonicated at 200 W for 15 minutes and then centrifuged at 4ºC, 30,000g for 15 minutes. The supernatant was mixed well with 5 × volume of chilled acetone containing 10% (v/v) trichloroacetic acid and incubated at −20ºC overnight. After centrifugation at 4ºC, 30,000g, the supernatant was discarded. The precipitate was washed thrice with chilled acetone. The pellet was air-dried and dissolved in lysis buffer. The suspension was sonicated at 200 W for 15 minutes and centrifuged at 4ºC, 30,000g for 15 minutes. The supernatant was then transferred into a separate tube. To reduce disulfide bonds in proteins in the supernatant, 10 mM dithiothreitol was added and incubated at 56ºC for 1 hour. Subsequently, 55 mM iodoacetamide was added to block the cysteines, incubated for 1 hour in the dark. The supernatant was mixed well with 5 × volume of chilled acetone for 2 hours at −20ºC to precipitate proteins. After centrifugation at 4ºC, 30,000g, the supernatant was discarded, and the pellet was air-dried for 5 minutes, dissolved in 500 μL 0.5 M triethyl ammonium bicarbonate (Applied Biosystems, Milan, Italy), and sonicated at 200 W for 15 minutes. Finally, samples were centrifuged at 4ºC, 30,000g for 15 minutes. The supernatant was transferred to a new tube and quantified using the Bradford kit (Bio-Rad, Hercules, CA, USA). The proteins in the supernatant were kept at −80ºC for further analysis.

### 2.4. Protein digestion

Total protein (100 μg) was removed out of each sample solution, and the protein was digested with Trypsin Gold (Promega, Madison, WI, USA) at the ratio of protein: trypsin = 30: 1 at 37ºC for 16 hours. After trypsin digestion, peptides were dried by vacuum centrifugation. Peptides were reconstituted in 0.5M triethyl ammonium bicarbonate.

### 2.5. LC-multiple reaction monitoring (MRM)-MS

Samples were digested as previously described and spiked with 50 fmol of β-galactosidase for data normalization. MRM analyses were performed using a QTRAP 5500 mass spectrometer (SCIEX, Framingham, MA, USA) equipped with an LC-20AD nano HPLC system (Shimadzu, Kyoto, Japan). The Mobile phase consisted of solvent A (0.1% aqueous formic acid) and solvent B (98% acetonitrile with 0.1% formic acid). Peptides were separated on a C18 column (0.075 × 150 mm column, 3.6 μm) at 300 nL/min and eluted with a gradient of 5% to 30% solvent B for 38 minutes, 30% to 80% solvent B for 4 minutes, and maintained at 80% for 8 minutes. For the QTRAP5500 mass spectrometer, a spray voltage of 2400 V, nebulizer gas of 23 p.s.i., and dwell time of 10 ms were used. Multiple MRM transitions were monitored using unit resolution for both the Q1 and Q3 quadrupoles to maximize specificity.

### 2.6. Protein identify

Skyline software was used to integrate the raw files generated by QTRAP 5500 (SCIEX, Framingham, MA, USA). We used the iRT strategy to define the chromatography of a given peptide against a spectral library. All transitions for each peptide were used for quantitation unless interference from the matrix was observed. Spiked β-galactosidase was used for label-free data normalization. We used the MSstats with a linear mixed-effects model. The *P* values were adjusted to control for FDR at a cutoff of 0.05. All proteins with a *Q* value < 0.05 and a fold change (FC) larger than 1.5 are considered significant.

### 2.7. Functional analysis

Gene ontology (GO) (http://www.geneontology.org/) was used to describe the attributes of differentially expressed genes. Eukaryotic orthologous groups (KOGs) (http://www.ncbi.nlm.nih.gov/COG/) were used to describe the functional classification of differentially expressed genes. Pathway analysis was performed to identify significantly enriched differentially expressed gene pathways based on the Kyoto Encyclopedia of Genes and Genomes (KEGG) database (http://www.genome.jp/kegg/). Fisher exact test was performed to analyze the overlap between the GO/KEGG annotation list and list of differentially expressed genes. *P* < .05 was applied to denote the significant enrichment of GO terms/pathways.

### 2.8. Screening of the diagnostic molecular markers

The public information of the gene expression omnibus (GEO) database (https://www.ncbi.nlm.nih.gov/geo/) was used as reference data, and the differently expressed proteins (DEPs) in our study were measured as quantities of expression in the samples of the GEO database. The *t* test was used to identify the differential expression, with a *P* value < .05, considered as significant difference. According to the true positive rate (sensitivity) and false positive rate (1—specificity) of each DEPs, plotting the receiver operating characteristic (ROC) curve.

### 2.9. Immune infiltration analysis

The Public information from the GEO database was used as reference data, and CIBERSORT software was used to calculate enrichment scores and estimate cell infiltration. The *t* test was used to construct a linear model and calculate *P* values for differential expression. *P* Value < .05 was used to identify significantly differentially infiltrating immune cell types.

### 2.10. Statistical analysis

For continuous variables, data with a normal distribution are presented as mean ± SD, and the differences between the 2 groups were evaluated using an independent-samples *t* test. The difference in sex distribution between the 2 groups was evaluated using the chi-squared test. All statistical analyses were performed using SPSS version 26.0 (IBM Corp., Armonk, NY, USA). Differences yielding *P* < .05 were considered statistically significant.

## 3. Results

### 3.1. Baseline characteristics of study participants

The clinical and demographic characteristics of patients with IMN and healthy controls in this study are summarized in Table 1. Compared with the controls, the levels of urinary red blood cells and proteinuria were significantly increased (all *P* < .05). There were no significant differences in average age, sex distribution, serum creatinine, blood urea nitrogen, uric acid, S plasma albumin, total cholesterol, triglyceride, low-density lipoprotein, serum IgA, serum IgG, serum IgM, serum C3, or serum C4 levels between patients and controls (all *P* > .05). According to Ehrenreich system, all the patients had stage II disease.

**Table 1:** Clinical baseline data of patients with IMN and healthy controls.

Characters	IMN	Controls	*P* value
N	5	5	
Age (yr)	51.80 ± 5.07	47.00 ± 5.00	1.507
Sex (male/female)	3/2	4/1	>.999
URBC (/HPF)	2.00 ± 1.00	0.60 ± 0.55	.025
Proteinuria (g/d)	1.47 ± 0.76	0.26 ± 0.15	.009
SCr (μmol/L)	74.18 ± 19.33	70.23 ± 13.94	.721
BUN (mmol/L)	5.65 ± 2.08	5.21 ± 1.77	.729
UA (μmol/L)	320.16 ± 22.61	311.55 ± 35.09	.657
Plasma albumin (g/L)	35.27 ± 10.44	42.95 ± 5.71	.187
TC (mmol/L)	5.34 ± 2.02	4.59 ± 1.35	.510
Triglyceride (mmol/L)	1.61 ± 0.41	1.18 ± 0.20	.064
LDL (mmol/L)	2.45 ± 0.56	2.23 ± 0.58	.543
Serum IgA (g/L)	1.72 ± 0.62	1.58 ± 0.55	.714
Serum IgG (g/L)	12.08 ± 4.14	10.81 ± 4.02	.636
Serum IgM (g/L)	0.79 ± 0.15	0.73 ± 0.20	.648
Serum C3 (g/L)	1.06 ± 0.13	1.11 ± 0.26	.714
Serum C4 (g/L)	0.25 ± 0.07	0.27 ± 0.12	.650
Ehrenreich stage	II		

BUN = blood urea nitrogen, HPF = high power field, IMN = idiopathic membranous nephropathy, LDL = low density lipoprotein, SCr = serum creatinine, TC = total cholesterol, UA = uric acid, URBC = urinary red blood cell.

*P* < .05 indicates statistical significance.

### 3.2. Laser microdissection of glomeruli

**Figure 2.**
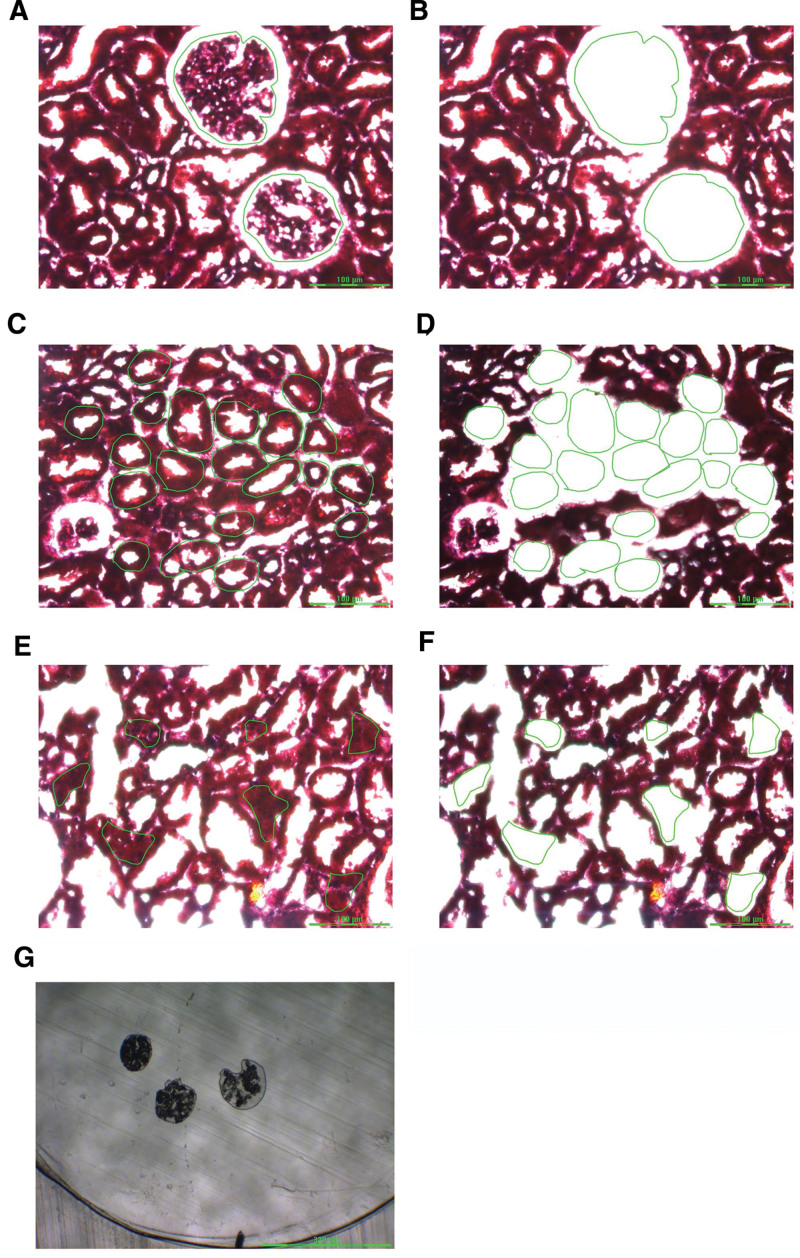
Laser microdissection of glomerulus in patients with IMN. (A) Glomerulus to be microdissected; (C) tubules to be microdissected; (E) interstitium to be microdissected; (B, D, and F) vacant space on slide following microdissection; (G) fragments of the microdissected glomerulus in the microcentrifuge tube cap. IMN = idiopathic membranous nephropathy.

Preserved glomeruli (five glomeruli per slide, 20 slides per case) were microdissected. Representative glomeruli, tubules, and interstitium of patients with IMN are shown in Figure 2. Figure 2A shows the marked glomerulus to be microdissected and Figure 2B shows the vacant area in the glomerulus after microdissection. Figure 2C shows the marked tubules to be microdissected, and Figure 2D shows the vacant area in the tubules after microdissection. Figure 2E shows the marked interstitium to be microdissected, and Figure 2F shows the vacant area in the interstitium after microdissection. Each small circle in the vacant area of the glomerulus, tubules, or interstitium represents 1 laser shot used to transfer the cut tissue into the tube cap. Figure 2G shows fragments of the glomerulus in microcentrifuge tube caps.

### 3.3. Identification of differentiated proteins

**Figure 3.**
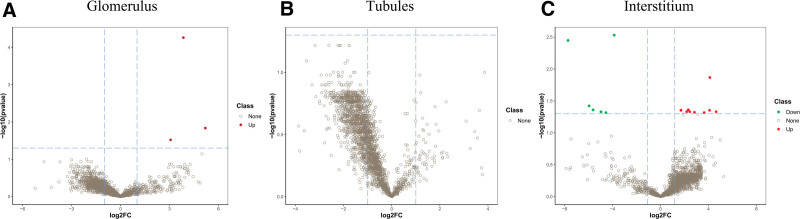
Altered expression profiles of proteins in kidney of patients with IMN. Volcano plot to exhibit 3 significantly changed proteins in glomerulus between IMN and control groups (FC > 1 and *Q *< 0.05) (A), no significantly changed proteins in tubules between IMN and control groups (FC > 1 and *Q *< 0.05) (B), and 15 significantly changed proteins in interstitium between IMN and control groups (FC > 1 and *Q *< 0.05). Red denotes up-regulated proteins and green denotes down-regulated ones. IMN = idiopathic membranous nephropathy.

A total of 2329, 2400 and 1850 proteins were identified in the renal glomeruli, tubules, and interstitium, respectively, in both groups. With a cutoff value of FC > 1 and *Q* value < 0.05, 3 upregulated proteins were differentially identified in the renal glomeruli between the IMN and control groups (Fig. 3A); 15 proteins (9 upregulated and 6 downregulated) were differentially identified in the renal interstitium between the IMN and control groups (Fig. 3C). However, no significantly downregulated proteins in the renal glomeruli or differentially expressed proteins in the renal tubules were identified in either group (Fig. 3B). In particular, complement factor H (CFH)-related protein 1 (CFHR1), immunoglobulin heavy constant gamma 3, and CFHR3 are upregulated in the renal glomeruli of patients with IMN. Coatomer subunit delta Archain 1 (ARCN1), putative small nuclear ribonucleoprotein G-like protein 15, and prostaglandin reductase 1 were the 3 most significantly upregulated proteins in the renal interstitium of patients with IMN; in contrast, fibrinogen gamma chain, CFH, and histidine-rich glycoprotein were the 3 most significantly downregulated proteins in the renal interstitium of patients with IMN (Tables 2 and 3).

**Table 2:** Comparison of up-regulated protein expression in the renal glomeruli in the renal glomeruli between the IMN group and the control group.

Protein ID	Protein	Regulation	FC	*Q* value
Q03591	Complement factor H-related protein 1	Up	29.149	2.285E-02
P01860	Immunoglobulin heavy constant gamma 3	Up	15.457	4.706E-05
Q02985	Complement factor H-related protein 3	Up	8.961	3.105E-02

IMN = idiopathic membranous nephropathy.

**Table 3:** Comparison of up- and down-regulated protein expression in the renal interstitium in the renal glomeruli between the IMN group and the control group

Protein ID	Protein	Regulation	FC	*Q* value
P48444	Coatomer subunit delta	Up	19.959	4.586E-02
A8MWD9	Putative small nuclear ribonucleoprotein G-like protein 15	Up	13.838	4.436E-02
Q14914	Prostaglandin reductase 1	Up	10.873	4.910E-02
P09382	Galectin-1	Up	6.064	4.769E-02
O75323	Protein NipSnap homolog 2	Up	4.709	4.586E-02
Q96TA1	Protein Niban 2	Up	4.586	4.436E-02
P62070	Ras-related protein R-Ras2	Up	4.439	4.586E-02
O15230	Laminin subunit alpha-5	Up	4.158	1.358E-02
P60981	Destrin	Up	3.258	4.436E-02
P02679	Fibrinogen gamma chain	Down	0.005	3.534E-03
P08603	Complement factor H	Down	0.016	4.044E-02
P04196	Histidine-rich glycoprotein	Down	0.020	4.586E-02
Q5T5C0	Syntaxin-binding protein 5	Down	0.033	4.769E-02
Q02985	Complement factor H-related protein 3	Down	0.043	4.769E-02
P01860	Immunoglobulin heavy constant gamma 3	Down	0.066	2.949E-03

IMN = idiopathic membranous nephropathy.

### 3.4. GO analysis of differentiated proteins

**Figure 4.**
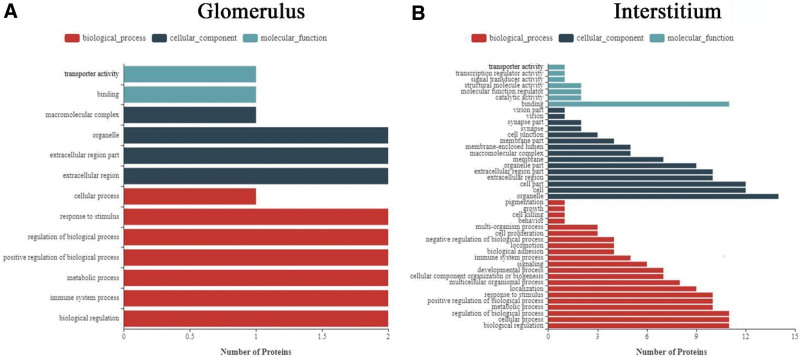
Differentially expressed proteins in glomerulus (A) and interstitium (B) characterized by GO classification. The bar graph shows the top ten enrichment scores values for the significant enrichment items. GO = gene ontology.

To further understand the function of the differentially expressed proteins, they were first analyzed based on the GO system. In the biological process analysis, the upregulated proteins in the glomeruli of patients with IMN were enriched in “transporter activity” and “binding” (Fig. 4A). In the cellular component analysis, proteins in the glomerulus were mostly enriched in the extracellular region and organelles (Fig. 4A). In molecular function analysis, proteins in the glomerulus were mostly enriched in “biological regulation,” immune system processes, positive regulation of biological processes, regulation of biological processes, and response stimuli (Fig. 4A). On the other hand, in biological process analysis, the different expressed proteins in interstitium of patients with IMN were mostly enriched in “bind” (Fig. 4B). In the cellular component analysis, proteins in the interstitium were mostly enriched in “organelles” (Fig. 4B). In the molecular function analysis, proteins in the interstitium were mostly enriched in “biological regulation,” cellular processes, and regulation of biological processes (Fig. 4B).

### 3.5. KEGG analysis of differentiated proteins

**Figure 5.**
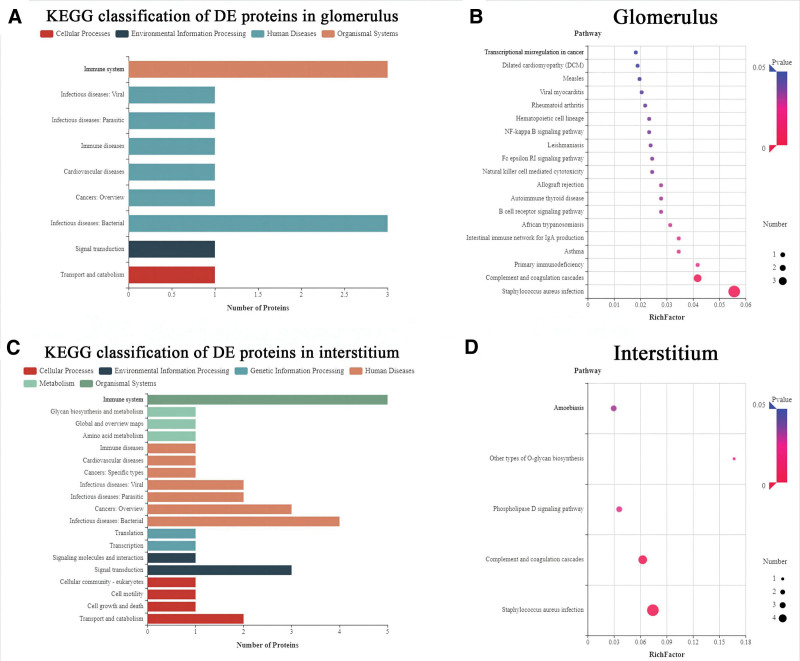
Differentially expressed proteins characterized by KEGG analysis. KEGG classification (A) and pathway analysis (B) of differentially expressed proteins in glomerulus; KEGG classification (C) and pathway analysis (D) of differentially expressed proteins in interstitium. KEGG = Kyoto Encyclopedia of Genes and Genomes.

KEGG classification analysis showed that, 3 up-regulation proteins in glomerulus of patients with IMN were mostly enriched in “Immune system” and “Infectious disease: Bacterial” (Fig. 5A). KEGG pathway analysis revealed that a total of 28 pathways were identified in glomerulus. The top 5 significant KEGG pathways were “*Staphylococcus aureus* infection,” “complement and coagulation cascades,” primary immunodeficiency, “Asthma” and “Intestinal immune network for IgA production” (Fig. 5B). On the other hand, KEGG classification analysis showed that, differentially expressed proteins in interstitium of patients with IMN were mostly enriched in “Immune system,” “Infectious disease: Bacterial,” “Cancer: Overview” and “Signal transduction” (Fig. 5C). KEGG pathway analysis revealed that a total of 50 pathways were identified in interstitium. The top 5 significant KEGG pathways were “*Staphylococcus aureus* infection, ” “Complement and coagulation cascades, ” “Phospholipase D signaling pathway, ” “Other types of O-glycan biosynthesis” and “Amoebiasis” (Fig. 5D).

### 3.6. Screening of the diagnostic molecular markers

**Figure 6.**
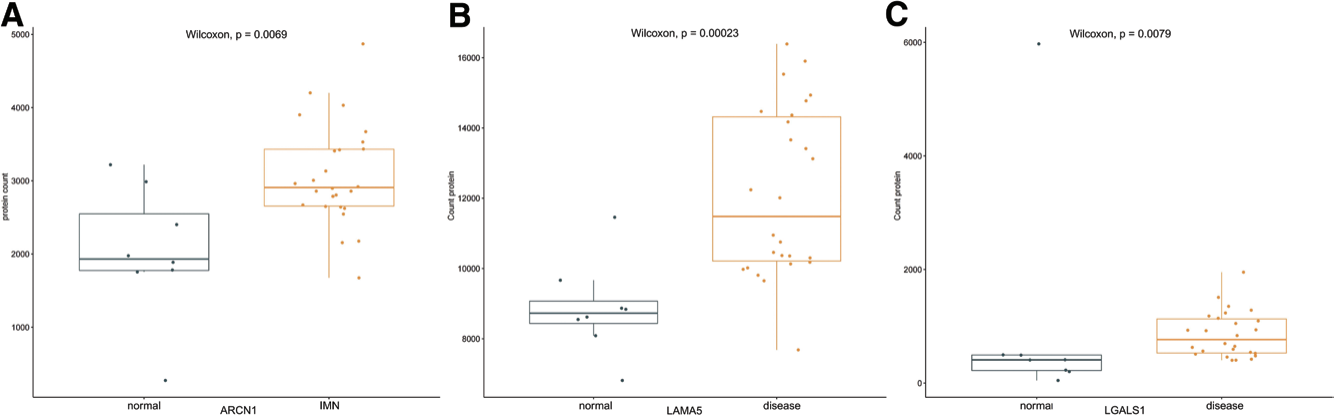
The counts of ARCN1 (A), LAMA5 (B), LGALS1 (C) expression in GEO database, orange nodes represent samples of IMN, green nodes represent samples of normal peoples. ARCN1 = Coatomer subunit delta Archain 1, GEO = gene expression omnibus, IMN = idiopathic membranous nephropathy, LAMA5 = Laminin subunit alpha-5, LGALS1 = Galectin-1.

**Figure 7.**
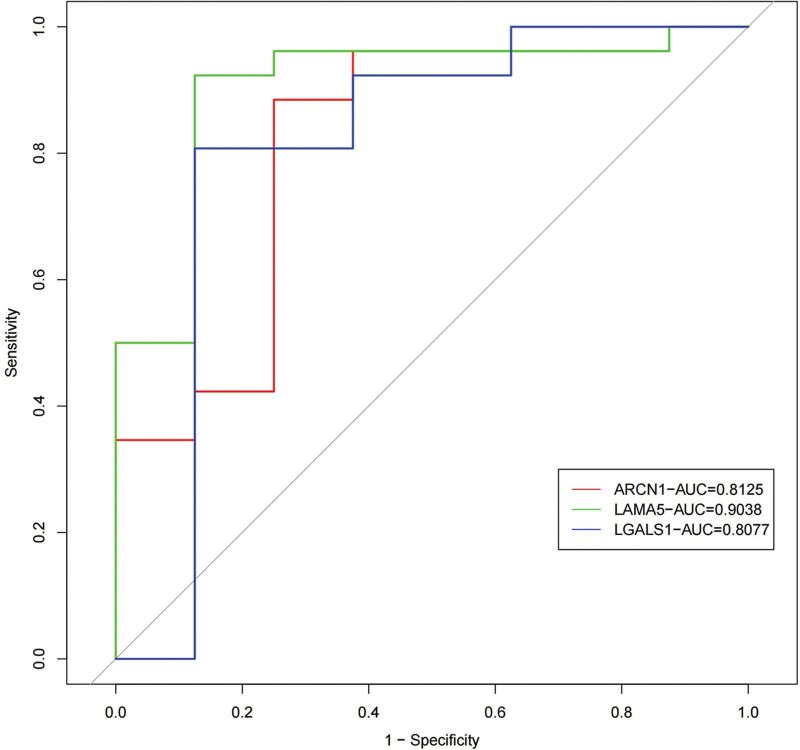
The ROC curves of ARCN1, LAMA5 LGALS1 in sensitivity of diagnosis IMN, red line represent ARCN1, green line represent LAMA5, blue line represent LGALS1, gray line represent control line (AUC = 0.5). ARCN1 = Coatomer subunit delta Archain 1, IMN = idiopathic membranous nephropathy, LAMA5 = Laminin subunit alpha-5, LGALS1 = Galectin-1, ROC = receiver operating characteristic.

A number of 8 normal glomerulus sample and 26 IMN glomerulus sample are found in GEO database. The DEPs in our study are measured quantity of expression in the samples of GEO database. The up-regulated protein in interstitium, ARCN1, Laminin subunit alpha (LAMA5), Galectin-1 (LGALS1) are significant highly expressed in IMN (*P* < .05). Protein expression levels are shown in Figure 6. The receiver operating characteristic curve was plotted to reflect the sensitivity of IMN diagnosis (Fig. 7).

### 3.7. Immune infiltration analysis

**Figure 8.**
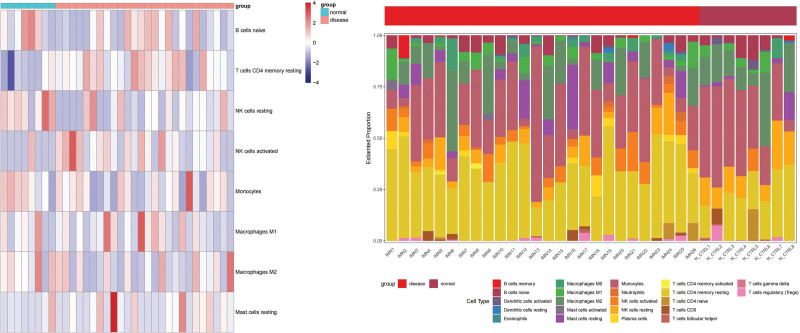
The heatmap (left) and bar plot (right) of immune cells proportion in the samples of GEO database. GEO = gene expression omnibus.

**Figure 9.**
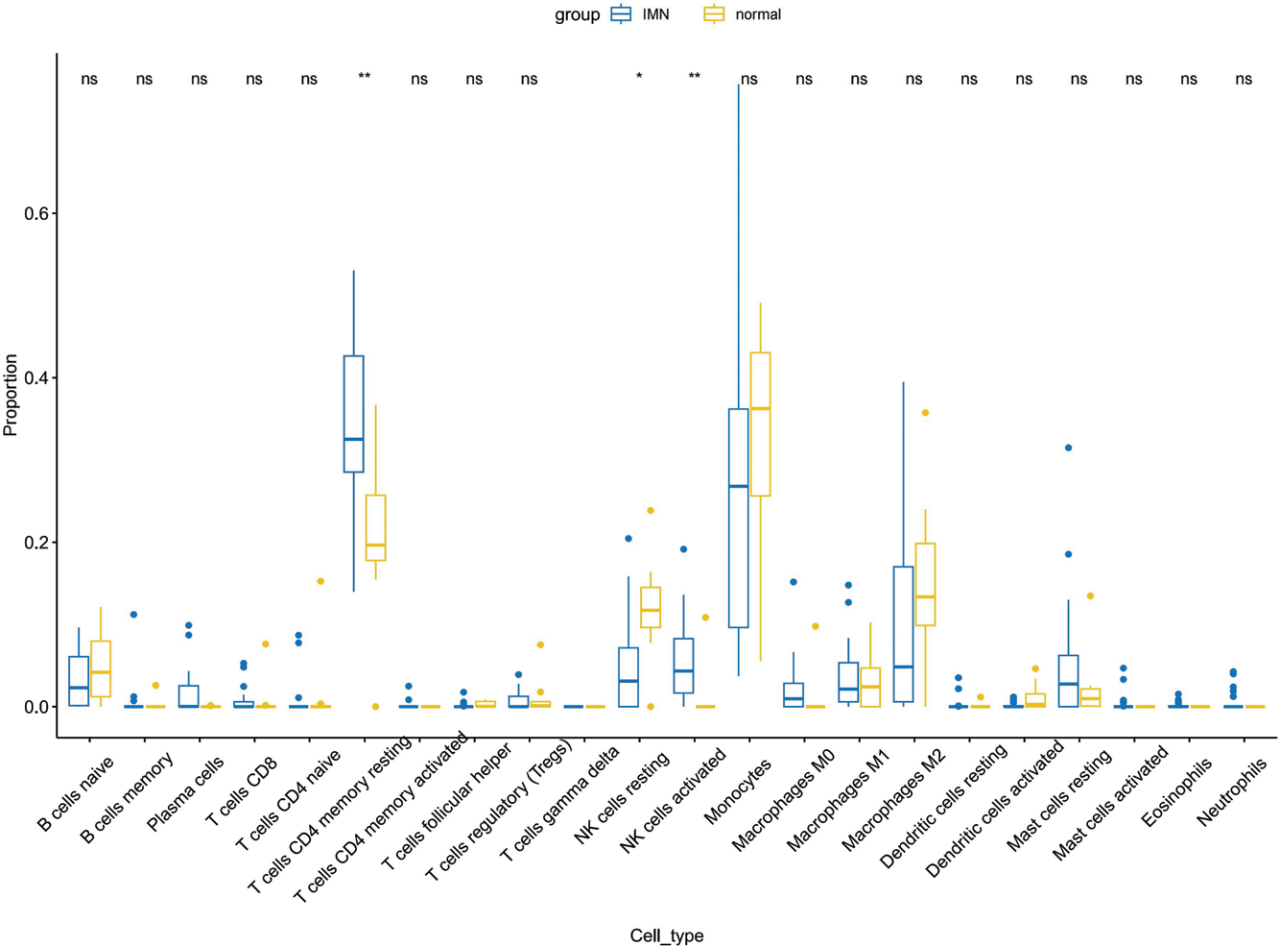
The box plot show different proportion of immune cells between IMN and normal control groups. Blue box represent IMN group, yellow box represent normal group. * represent significant different (*P* < .05). IMN = idiopathic membranous nephropathy.

CIBERSORT software was used to calculate enrichment scores and estimate immune cell infiltration in the samples from the GEO database. The expression of immune cells in each sample is shown as a functional heatmap and multi-panel bar plot (Fig. 8). The result show that the proportion of resting memory CD4 T cells and activated NK cells in IMN are significant higher than normal group, the proportion of resting NK cells are significant lower than normal group (*P* < .05), the result are summarized in a box plot (Fig. 9).

## 4. Discussion

IMN has a variable natural course. Some patients (approximately 20%–30%) may experience spontaneous remission, some persistently show proteinuria in the nephropathy or non-nephropathy category, and approximately 40% to 60% of patients may gradually progress to end-stage renal disease in 5 to 20 years.^[8,9]^ There are certain differences between studies regarding the relevant factors affecting the prognosis of IMN. Different studies have reached different conclusions regarding whether different types of pathological factors are related to the prognosis of patients with IMN^.[10,11]^ However, injury of tubular and interstitium, including tubule atrophy, interstitial fibrosis and inflammatory cell infiltration, etc were considered by most scholars to be closely related to the progression of renal function and prognosis.^[12–14]^ Therefore, exploring the immune mechanisms of different micro-regions of the kidney is of great significance for further elucidating the pathogenesis of IMN.

Previous studies have used LCM technology to classify and obtain mixed tissues, combined with LC-MS/MS or qRT-PCR technology to perform proteomic analysis of samples, thereby verifying the problem of tissue heterogeneity.^[15,16]^ In recent years, some scholars have used LMD combined with MS to analyze the proteome expression profile of kidney tissue slices, which improved the understanding of the pathogenesis of renal amyloidosis, membranous glomerulonephritis, and other kidney diseases, and improved the clinical diagnosis and classification methods.^[17,18]^ In this study, to explore the heterogeneity of different microregions of kidney tissue sections, we used LCM to successfully separate the glomeruli, renal tubules, and renal interstitium. Next, we used the LC-MRM-MS method to detect the protein expression profiles of glomeruli, tubules, and renal interstitium in patients with IMN and healthy controls. According to the results of the proteomic analysis in our study, although more than 1800 proteins were identified as co-expressed in the glomeruli, renal interstitium, and tubules between the 2 groups, compared with the control group, there were 3 significantly up-regulated proteins in the glomeruli of the IMN group, and was not a significantly down-regulated protein. In addition, compared with the control group, there were 9 proteins in the renal interstitium of the IMN group that were significantly increased, and 6 proteins were significantly down-regulated, and the protein expression of renal tubules in the 2 groups did not change significantly. These results indicate that pathophysiological changes in different microregions of the kidney tissue may be regulated by different signaling pathways. However, the number of differentially expressed proteins identified in the present study was relatively small. According to previous study,^[19]^ It is speculated that the reason may be related to our small sample size or the milder renal tubular lesions. However, these results provide a potential methodological and theoretical basis for exploring changes in the immune environment of renal tissue microregions.

With the aid of database retrieval technology, the functions of significantly differentiated proteins were preliminarily analyzed to discover the potential biological mechanisms of pathological changes in different micro-regions of kidney tissue. GO analysis showed that the differentially expressed proteins in the glomerulus and renal interstitium were enriched in biological regulation, the immune system, and metabolic processes. Furthermore, our study demonstrated that immunization predominantly involved the “immune system, ” and “complement and coagulation cascades” were enriched in the pathway and classification analysis of the glomerulus and renal interstitium. It has been clarified that IMN is induced by an underlying immune response.^[20]^ The immune complexes can lead to actin-cytoskeleton disorder and the disappearance of podocyte foot processes, which leads to podocyte damage, increased glomerular filtration barrier permeability, and ultimately to massive proteinuria and nephrotic syndrome.^[21]^ As it is well known that IMN is the pathological type of nephrotic syndrome associated with the highest incidence of thromboembolic events,^[22]^ however, the underlying mechanism of IMN-related hypercoagulability is not fully understood.^[23]^ Further efforts should be made to clarify the mechanism of action of the coagulation cascade in IMN-related thromboembolic events. These results suggest that these differentially expressed proteins may play a predominant role in immune responses.

CFH and CFHR1 are plasma proteins that play important regulatory roles in activation of the alternative complement pathway. CFH exerts an inhibitory effect on complement activation, whereas CFHR1 affects the inhibitory function of CFH by competitively binding to CFH surface ligands.^[24]^ A previous study indicated that because CFH is a major regulator of the alternative pathway, inhibition of CFH activity by autoantibodies at the podocyte cell surface might contribute to hyperactivation of the alternative pathway and accelerate disease progression, suggesting that a subset of patients with IMN have dysregulation of the alternative pathway.^[25]^ In addition, both CFHR1 and CFHR3 can inhibit the production of complement component 5a, thereby blocking the chemotaxis of neutrophils mediated by complement. They can also inhibit the binding of CFH and complement component 3b and compete with CFH for the binding site on complement component 3b.^[26]^ A previous study confirmed that variations in multiple loci of CFHR1 and CFHR3 are related to the risk of IgA nephropathy (IgAN). Plasma levels of CFHR1 in patients with IgAN are significantly higher than normal people.^[27]^ Knockout of CFHR1 and CFHR3 has a protective effect on IgAN.^[28]^ However, research on the relationship between CFH1 and IMN has not yet been conducted. On the other hand, LGALS1 has been discovered as a new protein that causes kidney fibrosis. Recent research showed that induction of kidney fibrogenesis through the accumulation of LGALS1 and cell matrix proteins by modulating protein synthesis in the kidney cortex of diabetic mice might play a major role in the pathological changes seen in DN, leading to renal failure.^[29]^

Due to the limited number of samples in our study, the DEPs in our study were compared to the public data in the GEO database; the up-regulated proteins ARCN1 LAMA5 LGALS1 in the interstitium of IMN are also upregulated in the glomerulus samples of the GEO database, suggesting that these proteins are differentially expressed between IMN and normal tissue, and may be potential biomarkers for diagnosing IMN. LGALS1, also known as LGALS1, is a protein that has been implicated in various immune regulations, current studies found that LGALS1 may promote the production of T helper 2 (Th2) cytokines and stimulate B cells to produce IgG4, which is a characteristic feature of IMN; LGALS1 expression in the glomeruli of patients with IMN has been associated with disease severity and poor prognosis. High levels of LGALS1 have been correlated with increased proteinuria, decreased renal function, and a higher risk of progression to end-stage renal failure. The result of our study agree these findings above. However there still no findings about the relationship between ARCN1, LAMA5 and IMN, further research is needed to verify.^[30]^

In order to explore the immune mechanism in IMN, we used immune infiltration analysis to explore the proportion of immune cells in IMN, based on the data in the GEO database and found that the proportion of resting memory CD4 T cells and activated NK cells in IMN is significantly higher than that in the normal group; however, the relationship between memory CD4 T cells and IMN is not explicitly mentioned in previous research, but there are indications that the altered functions of T cells. Memory CD4 T cells can differentiate into different subsets, including Th1 and Th2 cells. Th2 cytokines can stimulate B cells to produce IgG4 antibodies that characterize the immune response in IMN.^[31,32]^ However, there is no direct evidence that NK cells are involved in the immune mechanism of IMN. But it is possible that NK cells may indirectly affect the immune response in IMN through their interactions with other immune cells, such as T and B cells, and more evidence is needed for future research.

In summary, our current study has shown the protein expression of different microregions in the kidney tissue of patients with IMN through LCM combined with LC-MRM-MS analysis, bioinformatics analysis, and immune infiltration analysis, which revealed that there are significant differences in protein expression in different microregions of IMN. Although precise functional studies should be carried out in the future, our present study could provide a theoretical basis and a potential method for future research on the pathogenesis of IMN.

## 5. Limitation

Due to limited samples in our study, limited proteins are found meaningful to the pathogenesis of IMN. Deeply hope that there are more research data are shared in public data base, and the further research will be more meaningful. In addition, our study is a statistically observational study, we can’t give direct evidence to proof the protein contribute to pathogenesis of IMN, vitro experiment are needed in further research.

## Author contributions

**Conceptualization:** Chang Lu, Qiang Yan, Zhifeng Luo, Donge Tang, Fengping Zheng, Yong Dai.

**Data curation:** Chang Lu, Qiang Yan, Zhifeng Luo, Donge Tang, Fengping Zheng, Fanna Liu, Yong Dai, Weiguo Sui.

**Formal analysis:** Chang Lu, Donge Tang.

**Funding acquisition:** Qiang Yan, Fengping Zheng, Yong Dai, Weiguo Sui.

**Investigation:** Chang Lu, Qiang Yan, Zhifeng Luo, Shanshan Li.

**Methodology:** Chang Lu, Zhifeng Luo, Donge Tang, Shizhen Liu, Jing Qiu, Yong Dai, Weiguo Sui.

**Project administration:** Chang Lu, Shanshan Li.

**Resources:** Chang Lu, Zhifeng Luo, Shizhen Liu, Jing Qiu.

**Software:** Chang Lu, Donge Tang, Fengping Zheng, Shanshan Li, Shizhen Liu, Jing Qiu, Fanna Liu.

**Supervision:** Qiang Yan, Donge Tang, Jing Qiu, Yong Dai.

**Validation:** Fanna Liu.

**Visualization:** Chang Lu, Zhifeng Luo, Yong Dai, Weiguo Sui.

**Writing – original draft:** Chang Lu.

**Writing – review & editing:** Chang Lu.
